# Supplementary material for: Molecular architecture of the augmin complex
Source: Nat Commun. 2022 Sep 16;13:5449. doi: 10.1038/s41467-022-33227-7 (PMC9481612; doi:10.1038/s41467-022-33227-7)
Supplement: Supplementary file 1 — Supplementary Information [file 41467_2022_33227_MOESM1_ESM.pdf]

Supplementary Information

Molecular architecture of the augmin complex

Clinton A. Gabel[1,3], Zhuang Li[1,3], Andrew G. DeMarco[2,3], Ziguo Zhang[4], Jing Yang[4], Mark C. Hall[2,3], David Barford[4], and Leifu Chang[1,3,*]

[1]Department of Biological Sciences, Purdue University, West Lafayette, IN 47907, USA.

[2]Department of Biochemistry, Purdue University, West Lafayette, IN 47907, USA.

[3]Purdue University Center for Cancer Research, Purdue University, West Lafayette, IN 47907, USA.

[4]MRC Laboratory of Molecular Biology, Cambridge, CB2 0QH, UK.

*Correspondence to L.C. (lchang18@purdue.edu).

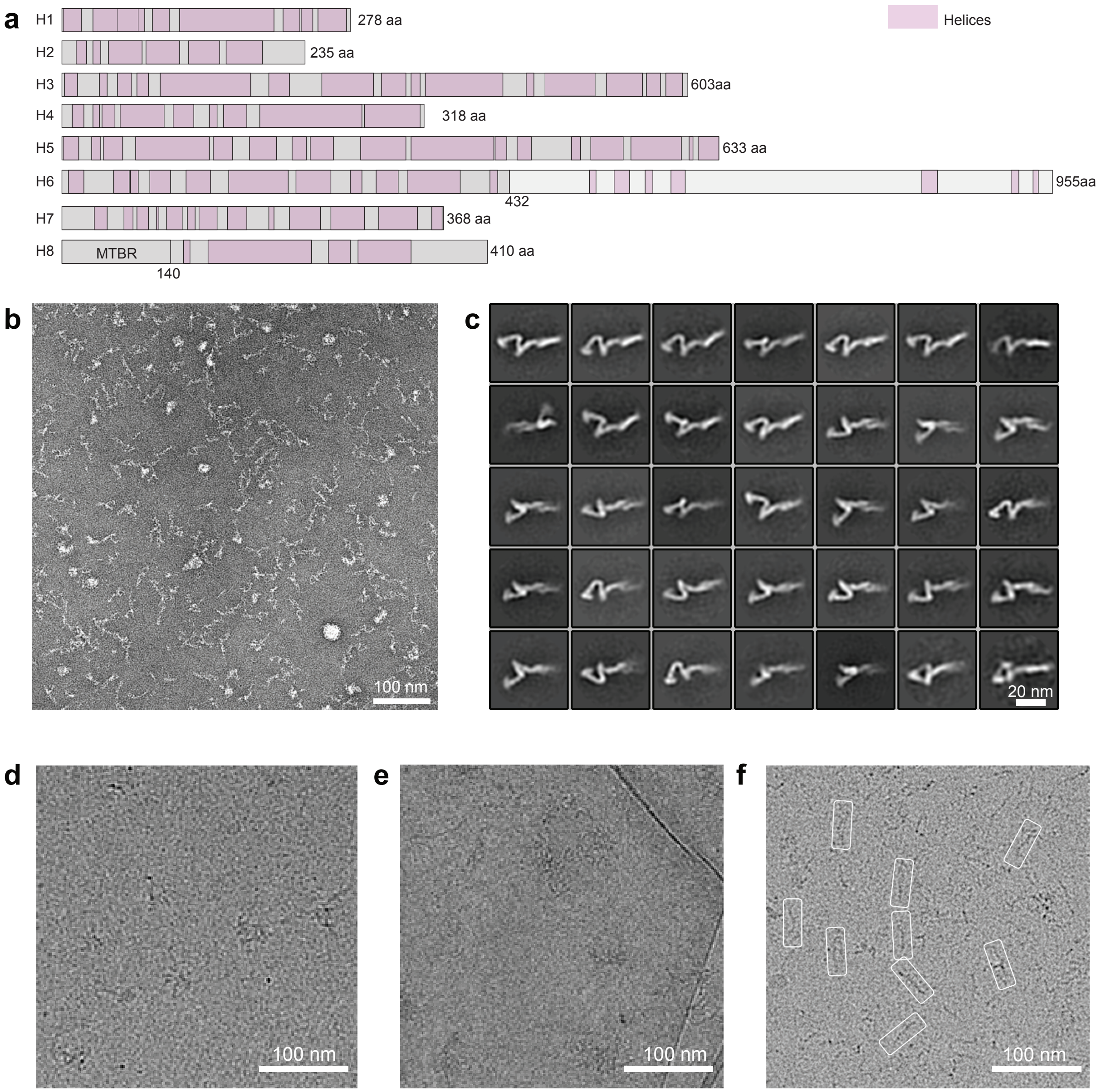

Supplementary Figure 1. **Secondary structure prediction and EM methodology. (a)** Schematic of HAUS subunits with predicted α-helices highlighted in pink. **(b)** Negative stain EM micrograph of augmin holo-complex from a total of 210 micrographs. Scale bar: 100 nm. **(c)** Representative 2D classes (out of a total of 100 classes) of augmin holo-complex from negative stain EM. Scale bar: 20 nm. **(d)** A cryo-EM micrograph of augmin$^{\Delta H6C}$ on holey carbon grids from a total of 20 micrographs. Scale bar: 100 nm. **(e)** A cryo-EM micrograph of augmin$^{\Delta H6C}$ on holey carbon grids covered with graphene oxide flakes from a total of 20 micrographs. Scale bar: 100 nm. **(f)** A cryo-EM micrograph of augmin$^{\Delta H6C}$ on holey carbon grids covered with a thin,

amorphous carbon layer from a total of 2,100 micrographs. Augmin particles are indicated in white boxes. Scale bar: 100 nm.

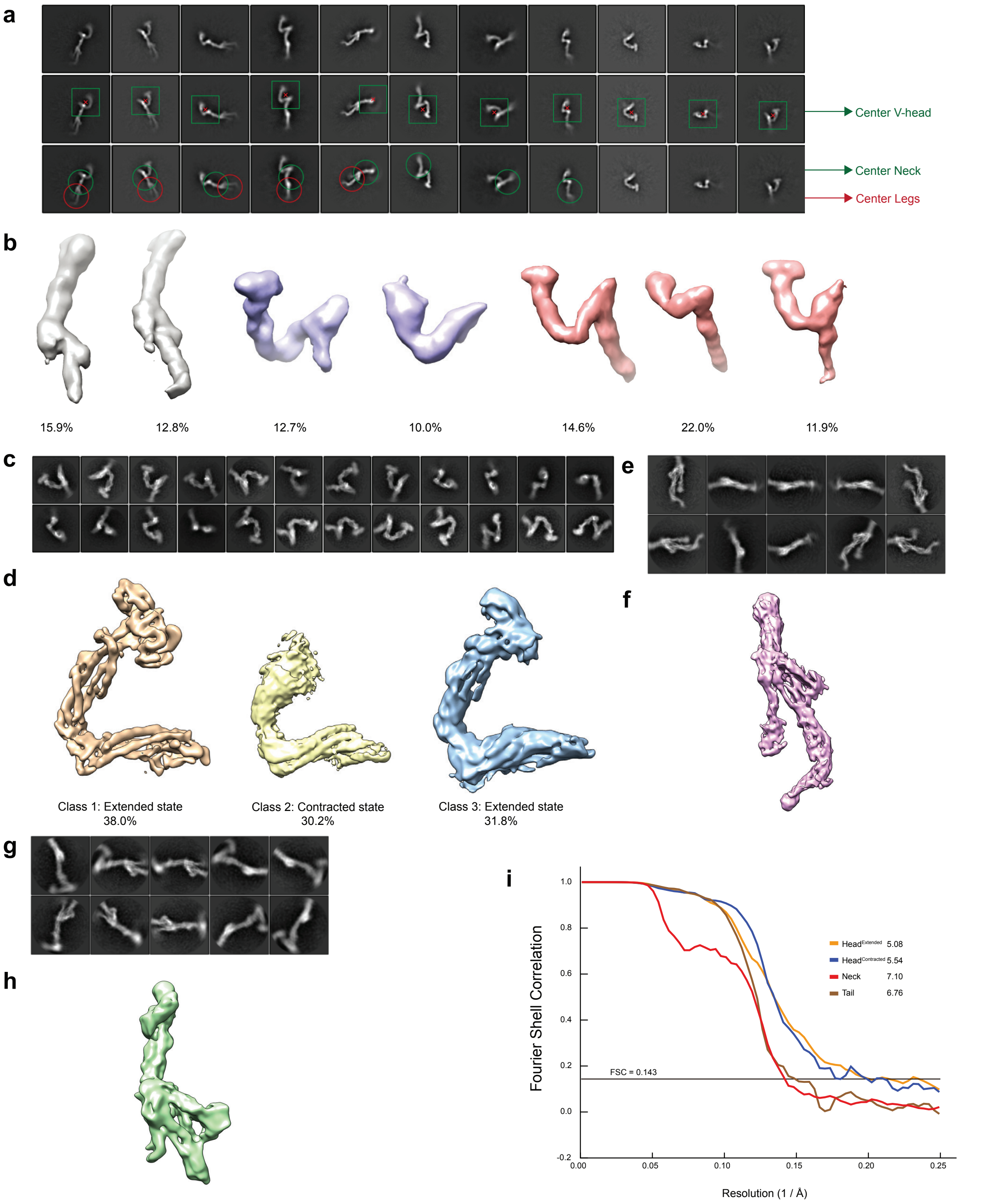

Supplementary Figure 2. Segmentation of augmin for cryoEM analysis. (a) Cryo-EM 2D classes of augmin$^{\Delta H6C}$. Green squares indicate classes centered on the V-shaped head. Green circles indicate classes centered on the neck. Red circles indicate classes centered on the legs of the tail. **(b)** Overall 3D classification maps of augmin. Percentage of total particles for each

volume listed below volumes. **(c)** 2D classes of the segmented V-shaped head. **(d)** Resulting 3D classification volumes from the 2D classes of the V-shaped head in **c**. Classes 1 and 3 represent the extended state of augmin. Class 2 represents the contracted state of augmin. **(e)** 2D classes of the centered legs. **(f)** Resulting 3D map of the legs from the 2D classes in **e**. **(g)** 2D classes of the centered neck of the tail. **(h)** Resulting 3D classification volume of the neck of the tail from the 2D classes in **g**. **(i)** FSC curves for the different maps represented in **d**, **f**, and **h**.

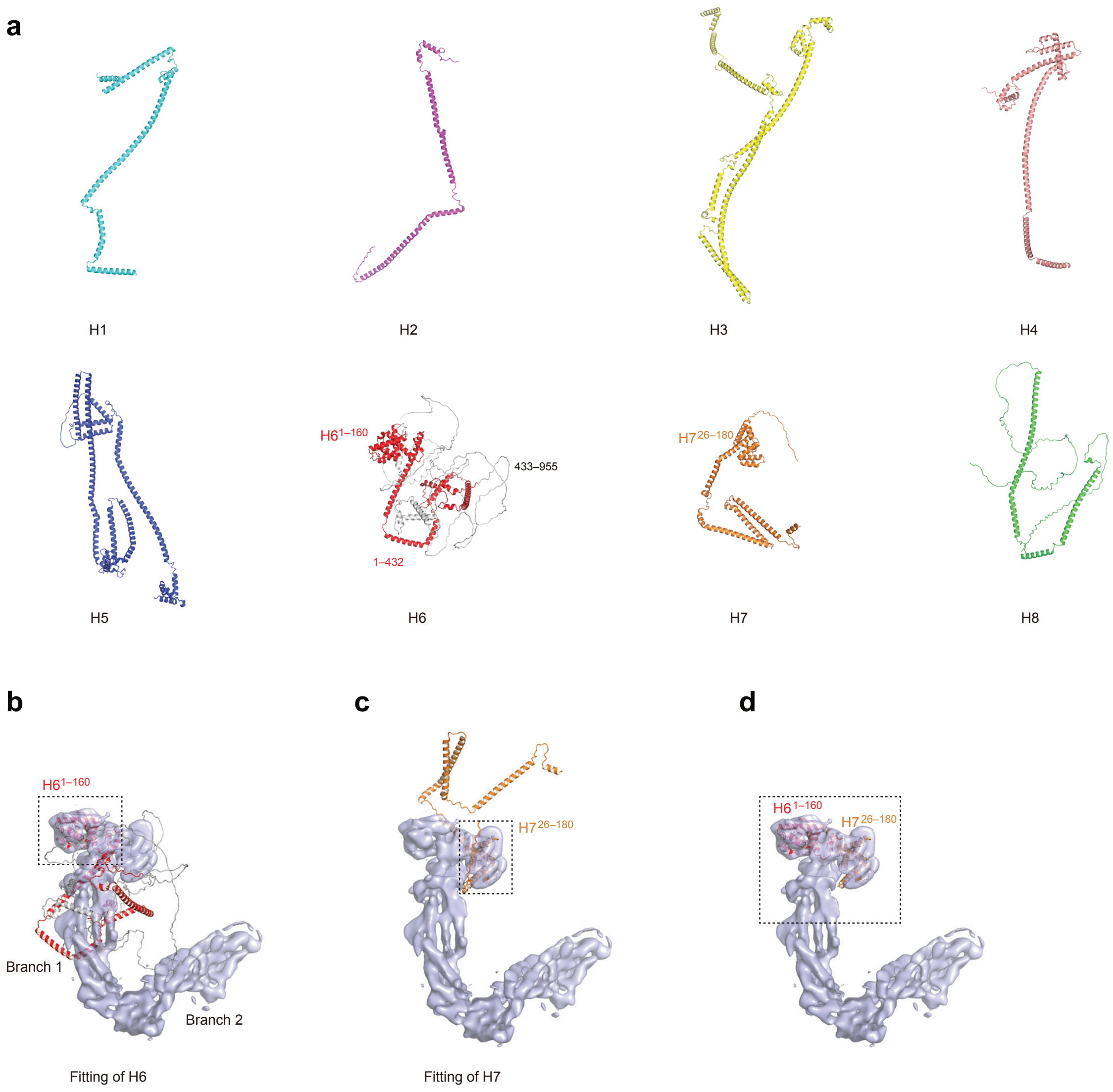

Supplementary Figure 3. Fitting of AlphaFold2 predictions into the tip of branch 1. (a) H1–8 subunits AlphaFold2 predictions from the AlphaFold Protein Structure Database. **(b)** Fitting of H6's AlphaFold2 structure prediction, a.a. 1–160, (α1–8) within the cryo-EM map of the tip of branch 1. **(c)** Fitting of H7's AlphaFold2 structure prediction, a.a. 26–180, (α1–8) within the cryo-EM map of the tip of branch 1. **(d)** Fitting of both N-termini of H6 and H7 within the cryo-EM map of the tip of branch 1.

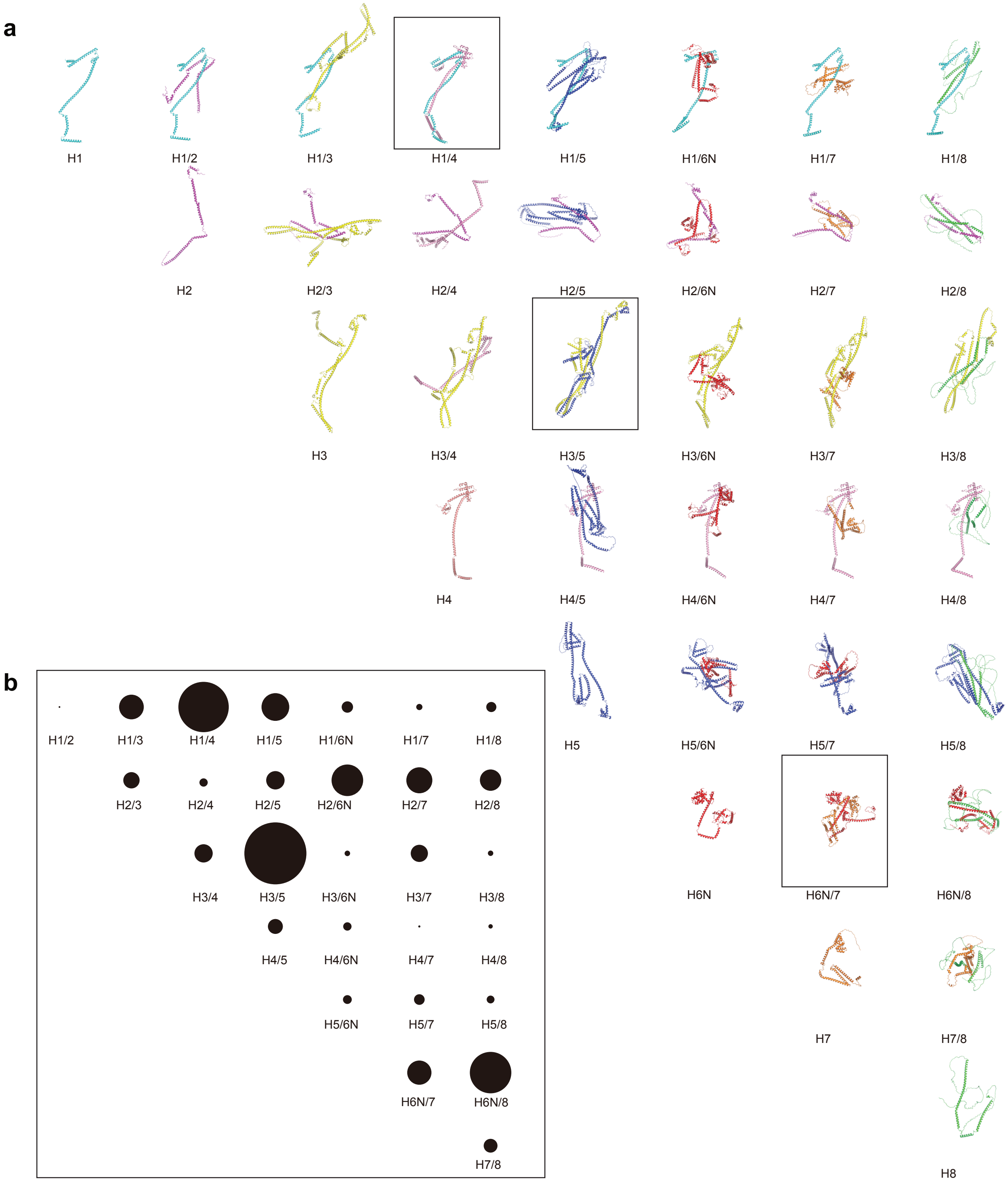

Supplementary Figure 4. Structure prediction of binary combinations of H1–8 subunits by ColabFold. (a) ColabFold prediction of binary combinations of H1–8 subunits. Binary combinations with structure well fitted within the cryo-EM map are indicated by boxes. **(b)** Representation of surface contact between subunits, with larger black circles representing greater contact between the two subunits.

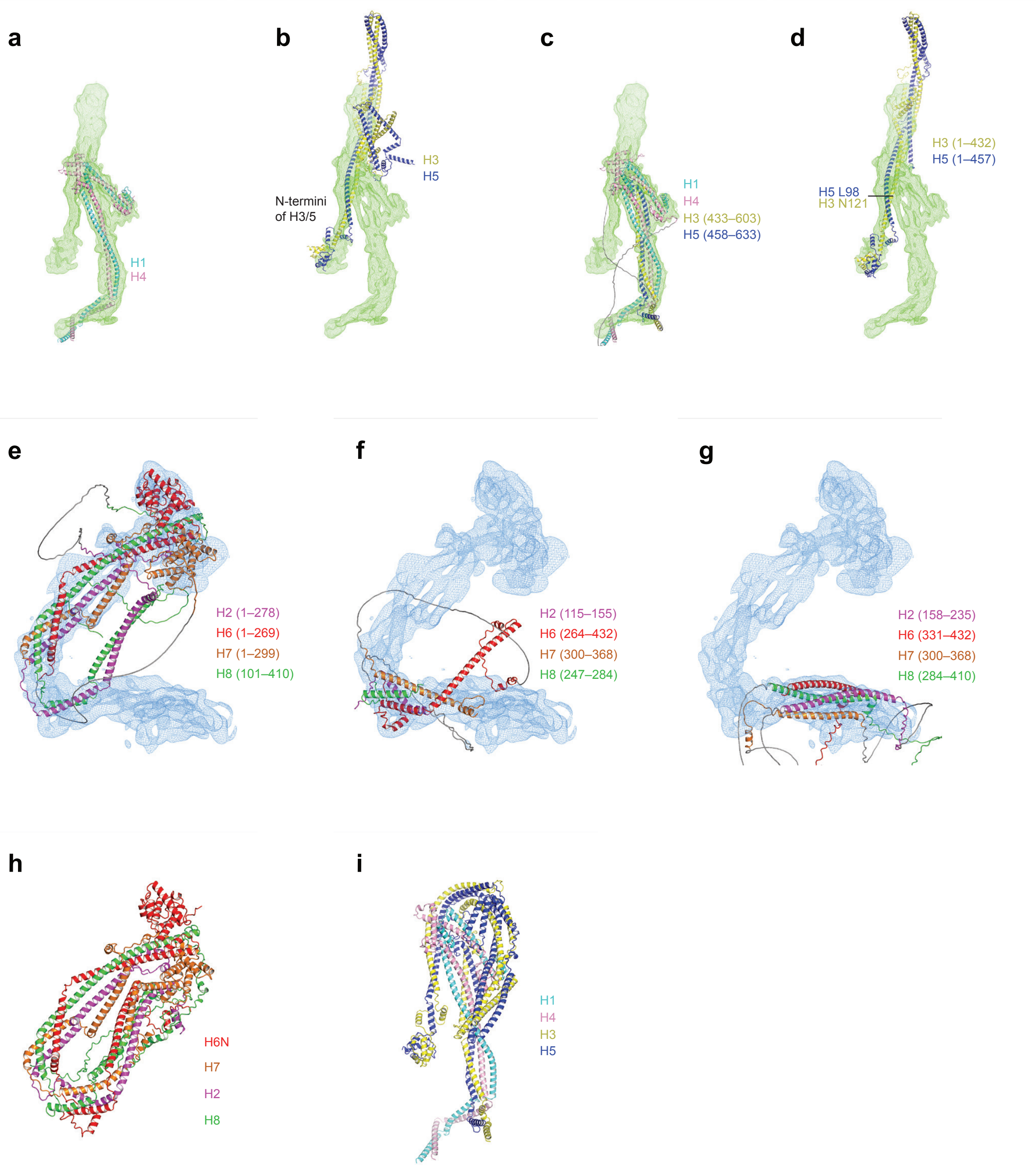

Supplementary Figure 5. Rigid-body fitting of predicted structure to the cryo-EM map of augmin. (a) Rigid-body fitting of the ColabFold predictions of the H1/4 dimer to the long leg in the cryo-EM map of augmin. **(b)** Rigid-body fitting of the ColabFold predictions N-termini of the H3/5 dimer to the short leg in the cryo-EM map of augmin. **(c)** Rigid-body fitting of the ColabFold predictions of the H1/4 dimer and the C-termini of the H3/5 dimer to the long leg in the cryo-EM map of augmin. 60 a.a glycine linkers were used to allow for structure prediction of more than two subunits. **(d)** Rigid-body fitting of the ColabFold prediction of the H3/5 dimer within the neck

and short leg of the cryo-EM map of augmin. **(e)** Rigid-body fitting of the ColabFold predictions of the N-termini of H2/6N/7/8 within branch 1 of the V-shaped head. 60 a.a glycine linkers were used to allow for structure prediction of more than two subunits. **(f)** Rigid-body fitting of the ColabFold predictions of H2/6N/7/8 within the kink of the V-shaped head. 60 a.a glycine linkers were used to allow for structure prediction of more than two subunits. **(g)** Rigid-body fitting of the ColabFold predictions of H2/6N/7/8 within branch 2 of the V-shaped head. 60 a.a glycine linkers were used to allow for structure prediction of more than two subunits. **(h)** AlphaFold Multimer prediction of the V-shaped head of augmin with H2/6N/7/8. **(i)** AlphaFold Multimer prediction of the tail of augmin with H1/3/4/5.

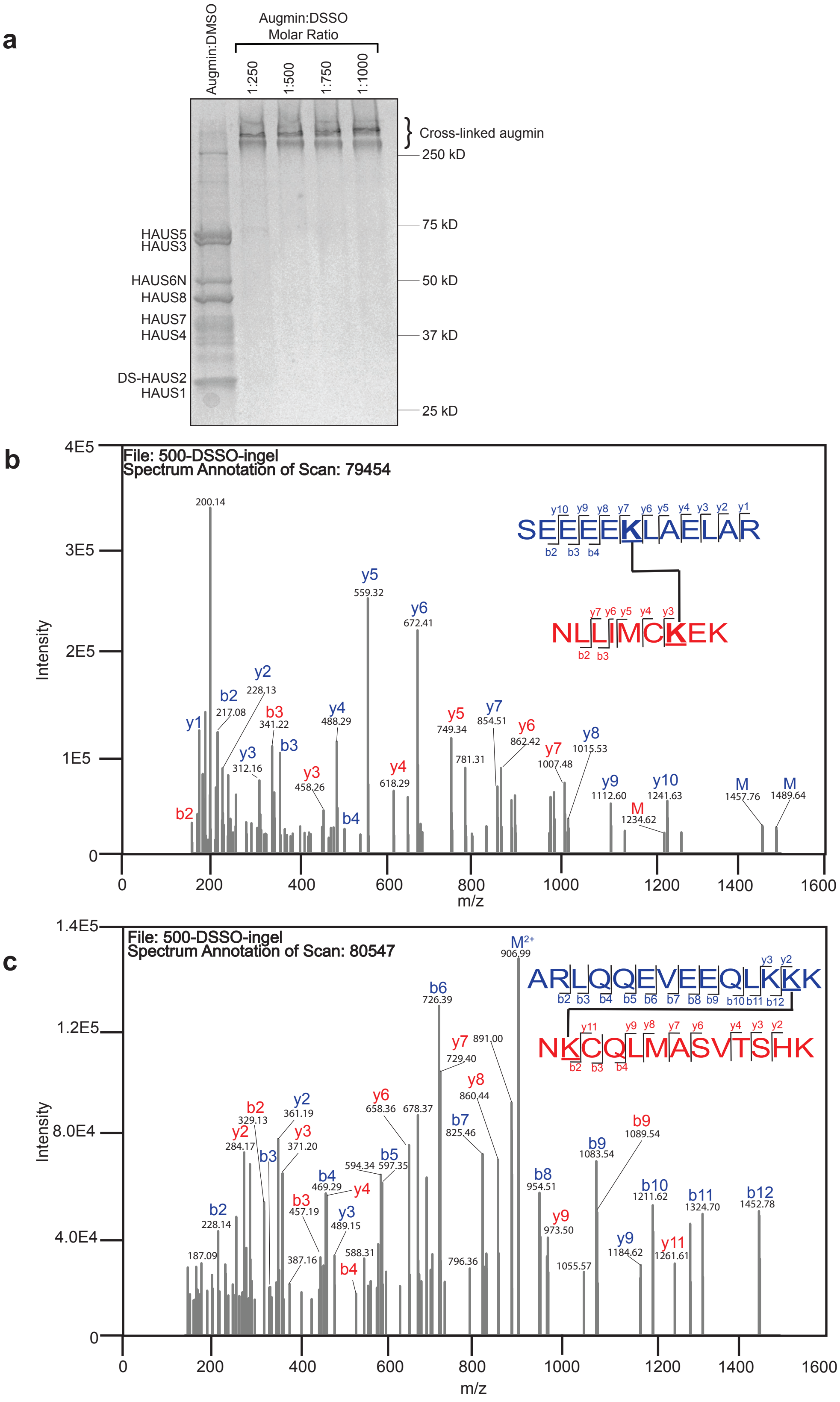
a
Augmin:DMSO
Augmin:DSSO
Molar Ratio
1:250
1:500
1:750
1:1000
Cross-linked augmin
250 kD
75 kD
50 kD
37 kD
25 kD
HAUS5
HAUS3
HAUS6N
HAUS8
HAUS7
HAUS4
DS-HAUS2
HAUS1
b
File: 500-DSSO-ingel
Spectrum Annotation of Scan: 79454
SEEEEKLAELAR
NLLIMCKEK
Intensity
m/z
c
File: 500-DSSO-ingel
Spectrum Annotation of Scan: 80547
ARLQQEVEEQLKKK
NKCQLMASVTSHK
Intensity
m/z

Supplementary Figure 6. Examples of MSMS spectra from identified DSSO-crosslinked HAUS peptides. (a) SDS-PAGE showing crosslinked augmin. The results shown are representative of more than three experiments. **(b,c)** y and b fragment ions and DSSO signature parent peptide ions (M) matched to HAUS subunit peptides by MetaMorpheus are labeled. Ions are singly charged unless indicated otherwise. **(b)** Peptide pair 26 from Table 3 containing HAUS7 peptide ^{219}SEEEE**K**LAELAR (blue labels) and HAUS8 peptide ^{177}NLLIMC**K**EK (red labels). **(c)** Peptide pair 7 from Table 3 containing HAUS4 peptide ^{116}ARLQQEVEEQL**K**K (blue labels) and HAUS3 peptide ^{121}N**K**CQLMASVTSHK (red labels).

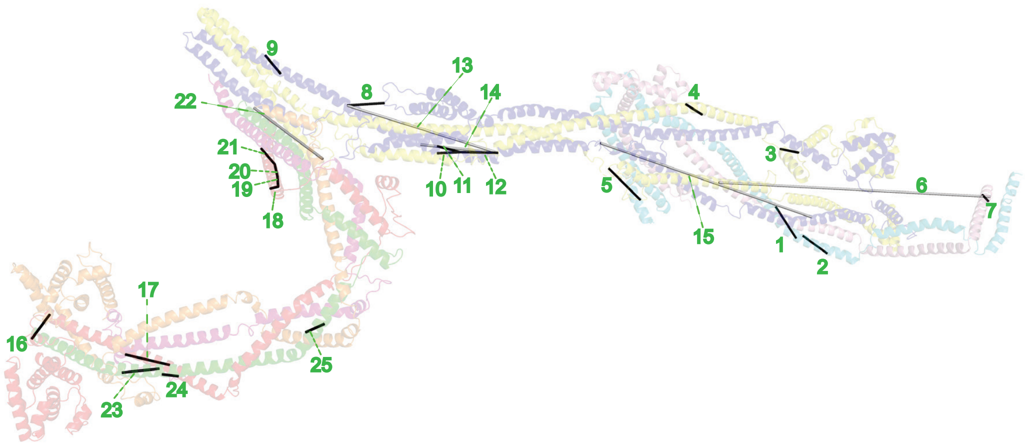

Supplementary Figure 7. Intrasubunit crosslinks identified in purified augmin$^{\Delta H6C}$ complex. Intrasubunit crosslinks are mapped onto the augmin structure using PyXlinkViewer. Subunits are colored as in other figures. Labels correspond to numbers in **Supplementary Table 2**. Black lines represent crosslink Cα-Cα distances <30 Å and light gray lines represent distances >30 Å.

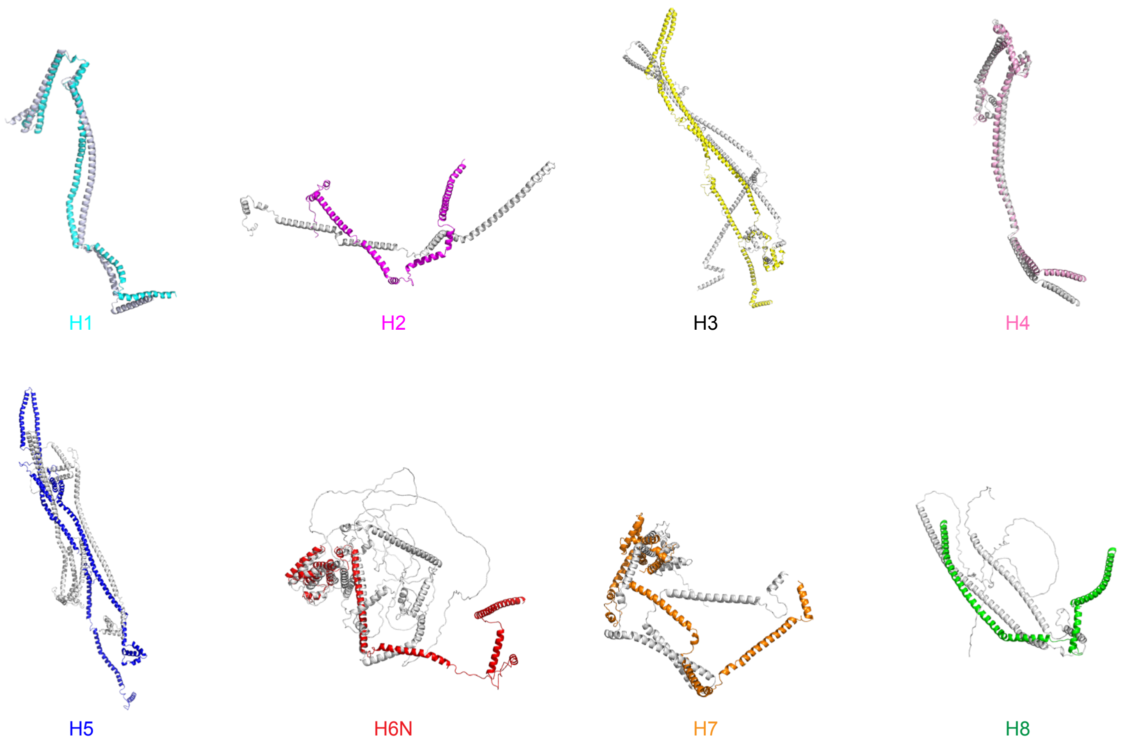

Supplementary Figure 8. Comparison of the cryo-EM map fitted subunits and AlphaFold2 Predictions. Cryo-EM map fitted HAUS subunits (colored) aligned with the single subunit predictions from the AlphaFold Protein Structure Database.

Supplementary Table 1 Cryo-EM data collection, refinement and validation statistics

	Head (Extended)	Tail	Neck	Head (Contracted)
**Data collection and processing**				
Magnification	105,000	105,000	105,000	105,000
Voltage (kV)	300	300	300	300
Electron exposure (e⁻/Å^2)	25	25	25	25
Defocus range (–μm)	0.6–1.2	0.6–1.2	0.6–1.2	0.6–1.2
Pixel size (Å)	1.384	1.384	1.384	1.384
Symmetry imposed	*C1*	*C1*	*C1*	*C1*
Initial particle images (no.)	1,173,445	1,173,445	1,173,445	1,173,445
Final particle images (no.)	447,864	218,459	71,325	355,252
Map resolution (Å)	5.08	6.76	7.10	5.54
FSC threshold	0.143	0.143	0.143	0.143
Map sharpening *B* factor (Å^2)	–400	–400	–120	–400
Map resolution range (Å)	5 – 8	6 – 10	7 – 11	5 – 9
**Refinement**				
Initial model used	ColabFold predictions			
Model resolution (Å)	9.4			
FSC threshold	0.5			
Model resolution range (Å)	5 – 20			
Model composition				
Non-hydrogen atoms	23600			
Protein residues	2927			
Nucleotides	0			
Ligands	0			
B factors (Å^2)				
Protein	312			
R.m.s. deviations				
Bond lengths (Å)	0.004			
Bond angles (°)	0.872			
**Validation**				
MolProbity score	1.55			
Clashscore	7.31			
Poor rotamers (%)	0.04			
Ramachandran plot				
Favored (%)	97.18			
Allowed (%)	2.79			
Disallowed (%)	0.03			

Supplementary Table 2. Intrasubunit crosslinks identified in purified augmin$^{\Delta H6C}$ complex.

	CL Distance (Å)	Protein	Peptide1	Peptide2	Score
1	18.2	HAUS1	^{179}RQNMDFL**K**AK	^{173}A**K**VDNR	15.5
2	14.1		^{194}FGI**K**AAEEQLSAR	^{187}A**K**SEEFR	18.5
	*	HAUS2	^{276}VHVQTINA**K**	^{253}ILAEESYLY**K**HDIIMPPLPFTSK	12.2
3	28.0	HAUS3	^{86}TSDL**K**TPR	^{67}SG**K**PILEGAALDEALKTCK	26.5
4	9.6		^{120}RN**K**CQLMASVTSHK	^{114}NL**K**IQR	16.2
	self-link		^{439}NTID**T**KDYSTHR	^{439}NTID**T**KDYSTHR	16.2
5	20.9		^{439}NTIDT**K**DYSTHR	^{451}LYQVLEGEN**K**KK	11.2
6	128.7	HAUS4	^{185}KSAAY**S**QVLLR	^{301}QATENKRWALQEF**S**KVYR	12.1
7	14.3		^{308}WALQEFS**K**VYR	^{301}QATEN**K**R	38.3
8	15.7	HAUS5	^{196}AQFLQNLLLPQA**K**R	^{378}ELQA**K**QQR	41.4
9	9.9		^{357}ALHDQ**S**QELQDAAGHR	^{349}CCLWTEL**K**	21.3
10	9.5		^{402}LLIKGNSA**S**KTR	^{417}**S**PGEVLALVQR	24.2
11	9.9		^{417}**S**PGEVLALVQR	^{406}GNSA**S**KTR	19.4
12	16.5		^{417}**S**PGEVLALVQR	^{428}**K**VVPTFEAVAPQSR	23.4
13	65		^{428}**K**VVPTFEAVAPQSR	^{378}ELQA**K**QQR	17.2
14	32.5		^{428}**K**VVPTFEAVAPQSR	^{402}LLI**K**GNSASK	25.5
15	109.2		^{565}ENLGQAL**K**R	^{574}LE**K**LLK	30.3
16	15.0	HAUS6	^{159}PQDLH**K**CIAR	^{146}NSSHHFVE**T**FNIK	11.2
17	18.5		^{190}YQENAQLSV**K**QVR	^{176}FLQILQRQDCV**T**QK	30.4
18	10.1		^{330}LTVDLHYLE**K**ETK	^{343}FQ**K**ER	11.1
19	10.7		^{348}LSDL**K**HMR	^{343}FQ**K**ER	21.4
20	10.4		^{348}LSDL**K**HMR	^{358}I**K**DDLTTIR	37.4
21	14.4		^{356}YRI**K**DDLTTIR	^{367}H**S**VVEK	13.2
22	39.3	HAUS7	^{335}AVETV**K**K	^{362}MNELME**K**	24.3
	self-link	HAUS8	^{12}PATGPTNSSSA**K**K	^{12}PATGPTNSSSA**K**K	10.1
	*		^{46}TTQ**K**APAGDGSQTR	^{39}YLQYE**K**K	34.4
	*		^{46}TTQ**K**APAGDGSQTR	^{60}G**K**MSEGGR	18.2
	*		^{46}TTQ**K**APAGDGSQTR	^{68}**K**SSLLQK	26.3
	*		^{60}G**K**MSEGGR	^{39}YLQYE**K**K	20.6
	self-link		^{60}G**K**MSEGGR	^{60}G**K**MSEGGR	16.4
	*		^{68}**K**SSLLQK	^{60}G**K**MSEGGR	29.5
	*		^{68}**K**SSLLQK	^{75}S**K**ADSSGVGK	23.5
	*		^{68}K**S**SLLQK	^{46}**T**TQKAPAGDGSQTR	14.1
	*		^{116}TPQLA**K**TISK	^{112}SIV**K**K	22.2
	*		^{126}**K**PESTSFSAPR	^{60}G**K**MSEGGR	12.5
	*		^{126}**K**PESTSFSAPR	^{116}TPQLA**K**TISK	23.5
23	14.1		^{173}RAE**K**NLLIMCK	^{184}E**K**EKLQKK	17.2
24	6.3		^{191}**K**AHELK	^{184}EKE**K**LQK	17.2
25	8.6		^{235}**T**FATALDTTRHELPVR	^{229}F**K**EQYR	16.1
	*		^{325}EAALANQEVWEE**T**QGMAPPSR	^{310}RSFAQVLELSAEA**S**K	24.4

Numbers in left column correspond to labels in Supplementary Fig. 7
*= peptide 1 is not present in structure
Crosslinked amino acids are underlined in bold
Score is the Total XL score from Metamorpheus.

Supplementary Table 3. Subunit names across species and NCBI accession IDs

H. sapiens	*D. melanogaster*	*A. thaliana*	*X. laevis*
HAUS1 isoform 1 (NP_612452.1)	Wee Augmin (WAC) (NP_001261194.1)	AUGMIN subunit 1 (NP_001031524.1)	HAUS1 (XP_041426206.1)
HAUS2 isoform 1 (NP_060567.1)	Msd1, Dgt9 (NP_612100.2)	AUGMIN subunit 2 (NP_565760.1)	HAUS2 (NP_001085195.1)
HAUS3 isoform 1 (NP_001290072.1)	Dgt3 (NP_01285856.1)	AUGMIN subunit 3 (NP_199663.2)	HAUS3 (NP_001086973.1)
HAUS4 isoform 2 (NP_001159742.1)	Dgt2 (NP_611533.2)	AUGMIN subunit 4 (NP_175486.2)	HAUS4 (NP_001089559.1)
HAUS5 isoform 1 (NP_056117.1)	Dgt5 (NP_001286349.1)	AUGMIN subunit 5 (NP_198704.2)	HAUS5 (XP_018082057.1)
HAUS6 isoform 1 (NP_060115.3)	Dgt6 (NP_65167.1)	AUGMIN subunit 6 (NP_568585.1)	HAUS6 (NP_001090564.1)
HAUS7 isoform 1 (Q99871.3)	Msd5, Dgt7 (NP_612101.1)	AUGMIN subunit 7 (NP_568354.1)	HAUS7 (NP_001121229.1)
HAUS8 isoform 1/A (NP_219485.1)	Dgt4 (NP_001259231.1)	AUGMIN subunit 8 (NP_001329921.1)	HAUS8 (NP_001090302.1)

Supplementary Table 4. Sequence comparison of human augmin subunits to other species.

H. sapiens HAUS Subunits	*D. melanogaster* (Identity/Similarity%)	*A. thaliana* (Identity/Similarity%)	*X. laevis* (Identity/Similarity%)
HAUS1	17.2/31.2	25.1/46.6	53.6/71.3
HAUS2	14.8/30.0	19.0/33.8	45.9/62.7
HAUS3	19.3/40.0	21.2/43.7	47.5/58.8
HAUS4	17.3/32.1	19.8/34.0	38.6/57.5
HAUS5	18.1/21.6	19.1/25.6	32.5/51.0
HAUS6	17.9/32.2	18.7/31.0	36.9/55.4
HAUS7	16.2/32.7	17.9/34.0	35.7/54.8
HAUS8	14.4/21.6	15.8/25.6	33.6/51.0

Supplementary Table 5. Oligonucleotide sequences for PCR amplification of H1–8.

Oligonucleotide	Sequences
H1_F	ATTTACAATCAtgGAGCCACAAGAGGAAAG
H1_R	ACCGCATGCtCACAACTCCATCATATCCACC
H2_F	ACTTCCAGTCAAtGGCAGCTGCAAACCCTTG
H2_R	AAGCTTGttaCTTTGCGTTAATGGTCTGAAC
H3_F	ATTTACAATCAtgTCATGTGGAAACGAATTTGTAG
H3_R	ACCGCATGCt CAATCTTCCAGGCTAACTGC
H4_F	ACCTATAAATAtgGCTTCAGGTGATTTCTGC
H4_R	AAGCTTGttaACGGTAG ACCTTAGAGAACT
H5_F	ACCTATAAATAtgGAATTAG CACAAGAAGCTC
H5_R	AAGCTTGtCAGCTGCACAGTTTCTGGA
H6_F	ATTTACAATCAtgTCAAGCGCATCTGTGAC
H6_R	ACCGCATGCttaCCTCGTA AGGTCGCTTG
H6N_F	ATTTACAATCAtgTCAAGCGCATCTGTGAC
H6N_R	ACCGCATGCttaATCAGGTAAACTGGCCGGGTATTGACA
H7_F	ATTTACAATCAtgGGAGGAGCTAGACTTGG
H7_R	ACCGCATGCttaCTTCTCCATCAGTTCGTTCA
H8_F	ACCTATAAATAtgGCTGATT CCTCCGGTAGAG
H8_R	AAGCTTGttaTGAAAGATCACGACCACTACGAGAGAGACT
