# Supplementary material for: Molecular architecture of the augmin complex
Source: Nat Commun. 2022 Sep 16;13:5449. doi: 10.1038/s41467-022-33227-7 (PMC9481612; doi:10.1038/s41467-022-33227-7)
Supplement: Supplementary file 3 — Description of Additional Supplementary Files [file 41467_2022_33227_MOESM3_ESM.pdf]

Description of Additional Supplementary Files

File Name: Supplementary Data 1
Description: Crosslinking mass spectrometry data showing the intra and intersubunit crosslinks, MetaMorpheus search parameters, and sequence coverage for the augmin subunits.

File Name: Supplementary Movie 1
Description: Extended and contracted state comparison at the head-tail connection. Movement of HB3 of the V-shaped head and HB4 of the neck of the tail undergoing a 20 Å conformational change when comparing the extended and contracted states.
