# Supplementary material for: Molecular architecture of the augmin complex
Source: Nat Commun. 2022 Sep 16;13:5449. doi: 10.1038/s41467-022-33227-7 (PMC9481612; doi:10.1038/s41467-022-33227-7)
Supplement: Supplementary file 6 — Reporting Summary [file 41467_2022_33227_MOESM6_ESM.pdf]

nature portfolio

Corresponding author(s): Leifu Chang

Last updated by author(s): Aug 15, 2022

Reporting Summary

Nature Portfolio wishes to improve the reproducibility of the work that we publish. This form provides structure for consistency and transparency in reporting. For further information on Nature Portfolio policies, see our Editorial Policies and the Editorial Policy Checklist.

Statistics

For all statistical analyses, confirm that the following items are present in the figure legend, table legend, main text, or Methods section.

n/a	Confirmed	
☐	☒	The exact sample size (*n*) for each experimental group/condition, given as a discrete number and unit of measurement
☒	☐	A statement on whether measurements were taken from distinct samples or whether the same sample was measured repeatedly
☒	☐	The statistical test(s) used AND whether they are one- or two-sided<br>*Only common tests should be described solely by name; describe more complex techniques in the Methods section.*
☒	☐	A description of all covariates tested
☒	☐	A description of any assumptions or corrections, such as tests of normality and adjustment for multiple comparisons
☒	☐	A full description of the statistical parameters including central tendency (e.g. means) or other basic estimates (e.g. regression coefficient) AND variation (e.g. standard deviation) or associated estimates of uncertainty (e.g. confidence intervals)
☒	☐	For null hypothesis testing, the test statistic (e.g. *F*, *t*, *r*) with confidence intervals, effect sizes, degrees of freedom and *P* value noted<br>*Give P values as exact values whenever suitable.*
☒	☐	For Bayesian analysis, information on the choice of priors and Markov chain Monte Carlo settings
☒	☐	For hierarchical and complex designs, identification of the appropriate level for tests and full reporting of outcomes
☒	☐	Estimates of effect sizes (e.g. Cohen's *d*, Pearson's *r*), indicating how they were calculated

Our web collection on statistics for biologists contains articles on many of the points above.

Software and code

Policy information about availability of computer code

Data collection	Leginon (J Struct Biol 151, 41-60)
Data analysis	Appion 3.3, Relion 3.0, Gctf 1.06, Motioncor2 1.1.0, PyMOL 2.2.3, Chimera 1.13, Coot 0.8.9.1, Phenix 1.18, cryoSPARC v1, ChimeraX 1.2.5, MetaMorpheus V0.0.320, xVis online server (https://xvis.genzentrum.lmu.de/login.php), PyXlinkViewer v3, Clustal Omega 1.2.1

For manuscripts utilizing custom algorithms or software that are central to the research but not yet described in published literature, software must be made available to editors and reviewers. We strongly encourage code deposition in a community repository (e.g. GitHub). See the Nature Portfolio guidelines for submitting code & software for further information.

Data

Policy information about availability of data

All manuscripts must include a data availability statement. This statement should provide the following information, where applicable:

- Accession codes, unique identifiers, or web links for publicly available datasets
- A description of any restrictions on data availability
- For clinical datasets or third party data, please ensure that the statement adheres to our policy

Cryo-EM reconstruction of the augmin complex has been deposited in the Electron Microscopy Data Bank under accession number EMD-25387. Coordinates for the atomic model of augmin have been deposited in the Protein Data Bank under accession number 7SQK. Mass spectrometry data has been deposited in the PRIDE Database under submission number PXD031411 [https://www.ebi.ac.uk/pride/archive/projects/PXD031411]. The source code of "rockstar.py" is available on GitHub [https://github.com/zhuangli200/Rockstar]. Single protein predictions for HAUS1–8 are available from the AlphaFold Protein Structure Database.

Human research participants

Policy information about studies involving human research participants and Sex and Gender in Research.

Reporting on sex and gender	NA
Population characteristics	NA
Recruitment	NA
Ethics oversight	NA

Note that full information on the approval of the study protocol must also be provided in the manuscript.

Field-specific reporting

Please select the one below that is the best fit for your research. If you are not sure, read the appropriate sections before making your selection.

☒ Life sciences ☐ Behavioural & social sciences ☐ Ecological, evolutionary & environmental sciences

For a reference copy of the document with all sections, see nature.com/documents/nr-reporting-summary-flat.pdf

Life sciences study design

All studies must disclose on these points even when the disclosure is negative.

Sample size	No sample-size calculation was performed. For cryo-EM, the actual sample size was increased until no further improvement in resolution was obtained in the cryo-EM maps.
Data exclusions	Cryo-EM particle images that fall into 2D averages or 3D classes with poor features were excluded from final reconstruction.
Replication	The crosslinking experiments were carried out as triplicates and reliably reproduced.
Randomization	Randomization is not relevant for the crosslinking experiments in this study. For cryo-EM, randomization was internally used by data processing software in initial particle assignment for 2D classifications and generation of half maps for resolution estimation.
Blinding	Blinding was not relevant to this study. Blinding is not typically performed for the crosslinking and structural studies reported in this paper.

Reporting for specific materials, systems and methods

We require information from authors about some types of materials, experimental systems and methods used in many studies. Here, indicate whether each material, system or method listed is relevant to your study. If you are not sure if a list item applies to your research, read the appropriate section before selecting a response.

Materials & experimental systems

n/a	Involved in the study	
x		Antibodies
	x	Eukaryotic cell lines
x		Palaeontology and archaeology
x		Animals and other organisms
x		Clinical data
x		Dual use research of concern

Methods

n/a	Involved in the study	
x		ChIP-seq
x		Flow cytometry
x		MRI-based neuroimaging

Eukaryotic cell lines

Policy information about cell lines and Sex and Gender in Research

Cell line source(s)	Sf9, clonal isolate derived from the parental Spodoptera frugiperda cell line IPLB-Sf-21-AE, Expression Systems, LLC Cat. # 94-001F<br>High Five™ (BTI-TN-5B1-4), ThermoFisher Cat. # B85502
Authentication	No cell lines were authenticated
Mycoplasma contamination	The cell lines were not routinely tested for mycoplasma contamination.
Commonly misidentified lines (See ICLAC register)	None
