# Supplementary figures and images for: Molecular architecture of the augmin complex
Source: Nat Commun. 2022 Sep 16;13:5449. doi: 10.1038/s41467-022-33227-7 (PMC9481612; doi:10.1038/s41467-022-33227-7)

# Source Data

Figure 1b

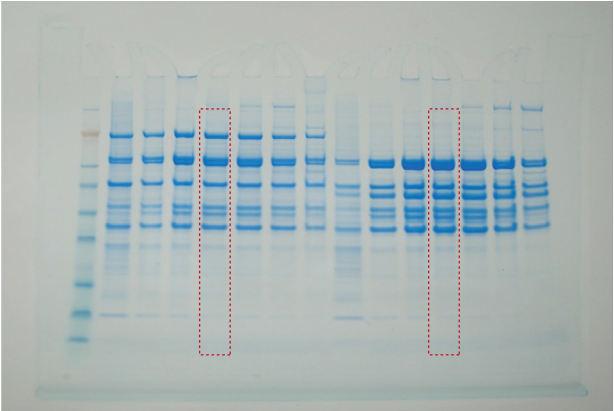

Supplementary Figure 6a

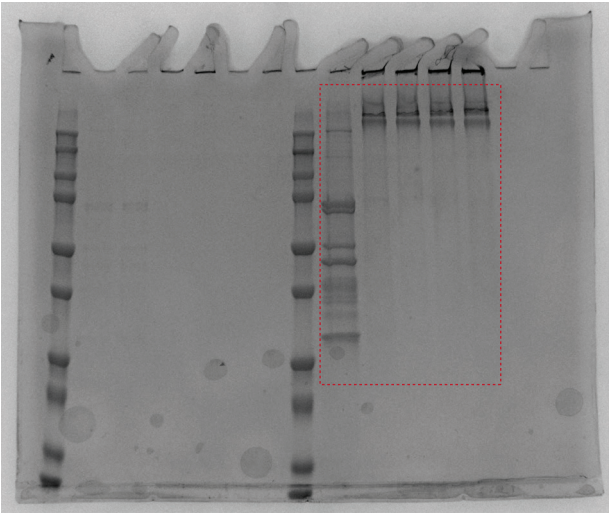

Supplement: Supplementary file 7 — Source Data [file 41467_2022_33227_MOESM7_ESM.pdf]
